# Assessment of Experimental Techniques That Facilitate Human Granuloma Formation in an In Vitro System: A Systematic Review

**DOI:** 10.3390/cells11050864

**Published:** 2022-03-02

**Authors:** Nirosha Ganesan, Steven Ronsmans, Jeroen Vanoirbeek, Peter H. M. Hoet

**Affiliations:** 1 Laboratory of Respiratory Diseases and Thoracic Surgery (BREATHE), KU Leuven, 3001 Leuven, Belgium; nirosha.ganesan@kuleuven.be (N.G.); jeroen.vanoirbeek@kuleuven.be (J.V.); 2 Centre for Environment and Health, Department of Public Health and Primary Care, KU Leuven, 3001 Leuven, Belgium; steven.ronsmans@kuleuven.be

**Keywords:** human in vitro model, granuloma, sarcoidosis, tuberculosis, schistosomiasis

## Abstract

The process of granuloma formation is complex, and due to species differences, the validity of animal studies is somewhat questioned. Moreover, the large number of animals needed to observe the different stages of development also raises ethical questions. Therefore, researchers have explored the use of human peripheral blood mononuclear cells (PBMCs), a heterogeneous population of immune cells, in an in vitro model. This review included in vitro studies that focused on exposing PBMCs—from healthy, sensitized, or diseased individuals—to antigens derived from infectious agents—such as mycobacteria or *Schistosoma* spp.—or inorganic antigens—such as beryllium. The reviewed studies mainly explored how human in vitro granuloma models can contribute towards understanding the pathogenesis of granulomatous diseases, especially during the early stages of granuloma formation. The feasibility of granuloma modelling was thus largely assessed via experimental techniques including (1) granuloma scoring indices (GI), (2) cell surface markers and (3) cytokine secretion profiling. While granuloma scoring showed some similarities between studies, a large variability of culture conditions and endpoints measured have been identified. The lack of any standardization currently impedes the success of a human in vitro granuloma model.

## 1. Introduction

Granuloma formation is a common histopathological feature of a range of infectious and non-infectious diseases [1,2]. A granuloma is a multicellular host-defense mechanism [3] initiated in response to foreign agents. It is assumed that its primary function is to contain an infectious agent by encapsulating it within a limited area to direct immune responses towards this region [4], hereby preventing the spread and enhancing the clearance of the agent [5]. Potential causative agents involved in granulomatous diseases are diverse and can be classified as infectious antigens, organic antigens, and inorganic particles. Infectious antigens are typically mycobacteria—such as *M. tuberculosis* and *M. leprae*—fungi, protozoa or parasites—such as *Schistosoma* spp. [6]. Organic antigens can be of plant or animal origin and are often associated with hypersensitivity pneumonitis [7]. Inorganic agents include particles such as silica, and metals such as beryllium (chronic beryllium disease), aluminium and titanium [6]. Sarcoidosis is a granulomatous disease with unknown etiology. Nevertheless, there is increasing evidence that sarcoidosis can occur in persons exposed to (combinations of) organic or inorganic agents [8].

The structure of an organized, well-defined granuloma involves a variety of immune cells—such as activated macrophages, dendritic cells (DCs), multinucleated giant cells (MGCs) or natural killer cells (NKs) [9] surrounded by a ring of T lymphocytes [10,11]. The formation of MGCs results from the fusion of macrophages and monocytes [12], and in most studies, MGCs are recognized as macrophages with two or more nuclei [5]. An increase in MGCs is seen as an indicator of increasing size of granuloma maturation. The lack of certain late-stage granuloma features such as epithelioid cells and increased MGC population can distinguish a well-defined granuloma from a structure encompassing only granuloma-like aggregates. 

Despite the large range of antigens involved in granuloma induction, most of the granulomas share a stereotypical structure determined by its cellular composition [13]. These similarities across granulomatous diseases propose that granulomas may depend on similar underlying mechanisms [14]. 

**Figure 1:**
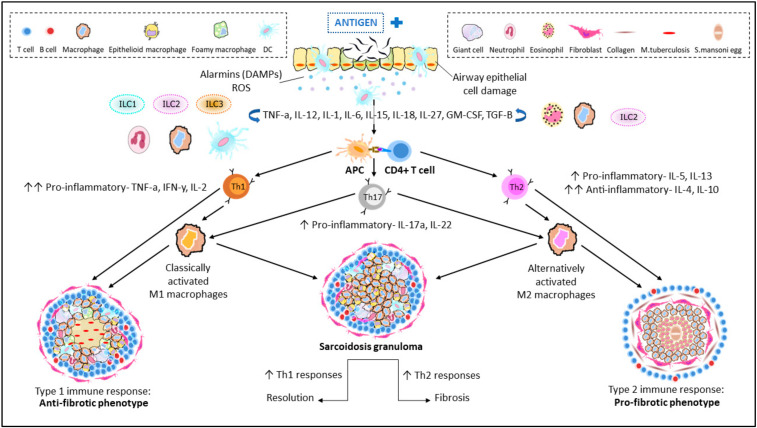
Immunopathogenesis of granulomatous diseases of the lung. Entry of an unknown airborne antigen simultaneously activates interstitial dendritic cells (DCs) and alveolar macrophages (AM), resulting in the eventual cellular damage of airway epithelial cells. Cellular damage induces the secretion of alarmins; a subgroup of damage-associated molecular patterns (DAMPs) and reactive oxygen species (ROS) that triggers the continuous secretion of key cytokines: TNF-α and IFN-γ and differentiation of CD4^+^ T cells (Th0). Upon antigen presentation to Th0, the host responds by differentiation and clonal expansion of Th1/Th2/Th17 subsets. Production of respective cytokines for Th differentiation is also triggered with the help of circulating macrophages, innate lymphoid cells (ILCs) and DCs to produce effector cytokines. Persistent stimulation and cellular recruitment over time eventually leads to formation of granulomas. A Th1-driven response leads to an anti-fibrotic granuloma phenotype, resembling a typical TB granuloma with its macrophage-rich core. On the other hand, Th2 cytokines contribute towards a pro-fibrotic granuloma with a characteristic collagen-rich exterior and eosinophilic core. An unstable balance between Th1 and Th2 is also able to promote resolution (Th1-driven) and fibrosis (Th2-driven) in sarcoid granulomas in humans. The role of Th17 cytokines: IL-17a and IL-22 is also well documented in both pathways.

Upon antigen presentation [15], CD3^+^CD4^+^ helper T cells (Th0) can be differentiated into several subsets of effectors cells, known as Th1, Th2, Th17 and Tregs. The presence of a majority of Th1 or Th2 cells (Figure 1) characterizes subsets of granulomatous diseases: Th1 cells, which are usually involved in the activation of M1 macrophages in mycobacterial infections, and Th2 cells, which are implicated in suppressing chronic inflammation [15]. Not only Th1/Th2 differentiation but also Th17 and Tregs—also recognized for mediating tissue inflammation and granuloma formation—play important roles in the phenotype of granulomas formed [16]. In the lung, granulomas can be further distinguished by the presentation of specific cell types [17] such as foreign body giant cells (in foreign body granulomas), epithelioid cells (ECs; in immune granulomas seen in sarcoidosis and tuberculosis) or Langerhans’ cells (histiocytosis X) [18]. 

In sarcoidosis, both Th1 and Th2 cells are identified, although it is unclear how their expression is balanced [19]. It is speculated that an upregulated Th1 response may result in a possible resolution of granulomas often observed in mycobacterial granulomas, whereas increased Th2 expression may resort towards a fibrotic phenotype, as observed in Th2-dominated *Schistosoma* granulomas [19]. This, however, requires further confirmation. 

Distinguishing protective and damaging mechanisms involved in granuloma formation per antigen, however, can prove to be vital in our understanding of granulomatous diseases [9,20]. In the course of granuloma maturation, end stages such as disintegration and caseous necrosis of granuloma structures have also been identified [10], although rarely studied in in vitro models.

To elucidate the mechanisms involved in granulomatous diseases, laboratory models are needed since animal models do not always recapitulate all aspects of any disease [21]. In recent years, in vitro granuloma modelling, using PBMCs of diseased and healthy individuals has garnered increasing interest (i.e., 13 studies in 2020–2021). 

Hence, in this review, we intend to explore the literature focused on in vitro models of (human) granuloma formation in the presence of an antigen or foreign compound (e.g., silica particles). 

The experimental setup and preference for specific techniques across different models will be reviewed to identify the models that have succeeded in inducing and maintaining granulomas in vitro.

## 2. Methodology

The review was conducted in accordance with the Preferred Reported Items for Systematic Reviews and Meta-analyses (PRISMA 2020 statement) [22]. The search strategy included studies published in PUBMED and EMBASE using the keywords “in vitro” AND “granuloma” (MeSH Terms). Only original studies written in English language and available in full-text format were selected. 

The inclusion criteria strictly adhered to studies with abstracts and/ or detailed methodology that address granuloma development in an in vitro setting using human peripheral blood mononuclear cells (PBMCs). 

**Figure 2:**
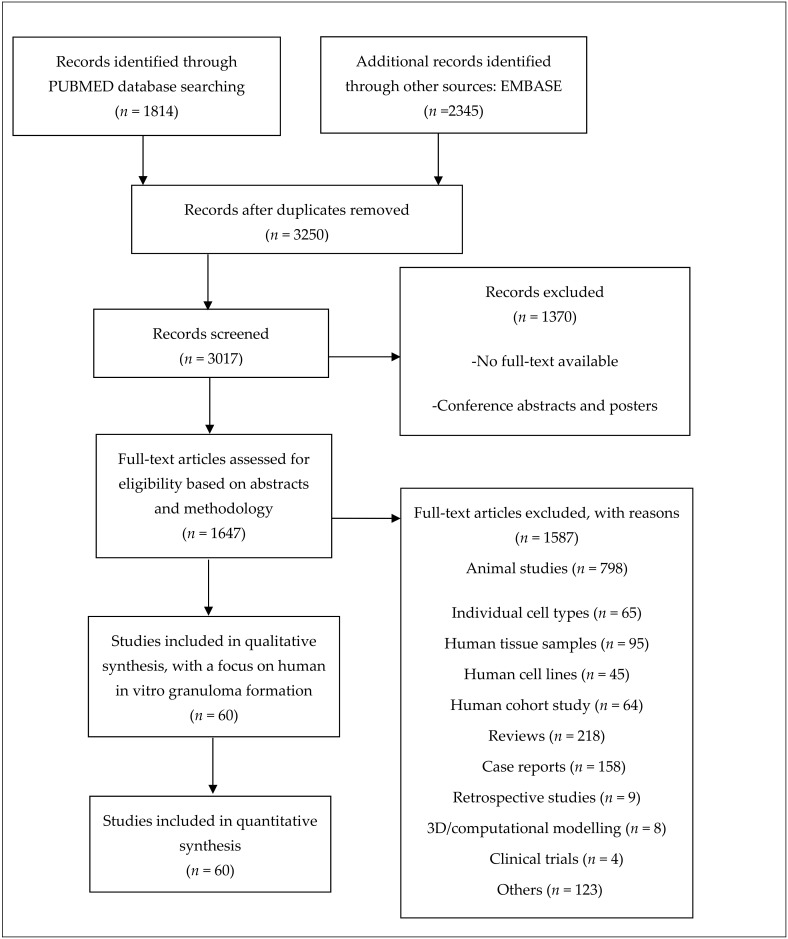
Workflow of literature review. Studies included were selected based on the following criteria: (1) a focus on in vitro granuloma modelling with human-derived peripheral blood mononuclear cells (PBMCs) and (2) successful granuloma development with an agent of choice. Other studies that focused on individual cultures of cells from PBMCs were excluded to avoid contradictions in the analyses. An overview of the included studies can be found as part of the Supplementary Materials.

Studies that focused on (i) animal studies, (ii) individual cell types, (iii) human tissue samples, (iv) human cell lines, (v) human cohort studies, (vi) reviews, (vii) case studies, (viii) retrospective studies, (ix) 3D/computational modelling and (x) clinical trials were not considered. Additionally, studies using cell lines or monocyte-derived macrophage (MDM) cultures, studies that focused on acute (1 day) rather than long-term exposure, and those that did not introduce exposure to at least one antigen or a foreign compound to evaluate granuloma development were excluded from this review (Figure 2).

## 3. Results

### 3.1. General Overview of the Included Studies

Sixty studies—oldest published in 1968 and most recent in 2021—were selected and included in this review. 

A breakdown of the types of agents/foreign compounds that have been used across the selected studies for this review is included (Table S1). Most studies used mycobacterial (65%) and *Schistosoma* (13%) antigens as inducing agents. 

In general, PBMCs came from three different types of populations: (1) healthy persons; (2) persons sensitized to an antigen due to previous infection or vaccination—e.g., with Bacillus Calmette-Guérin (BCG); and (3) persons with tuberculosis (TB), schistosomiasis or sarcoidosis. In 40% of studies, both healthy and individuals with diseases were included, while 10% only focused on individuals with diseases. 

The granuloma structures were confirmed by microscopy or flow cytometry, while cytokine profiling provided an understanding of the cellular interactions involved within and between different subsets of cells during the genesis of granulomas. Additionally, a handful of studies used scoring systems to assess the different stages of granuloma development over time.

In the following sections, we describe I. granuloma scoring indices, II. cell surface markers profiling (microscopy and flow cytometry) and III. cytokine secretion profiling and decipher how each technique contributes towards understanding granuloma development. 

### 3.2. Granuloma Indices

A numerical scoring system was introduced as early as 1987 (further indicated as GI-B, Table 1) to distinguish between different stages during development of granulomas. Studies that made use of this scoring index (89% of studies) used *Schistosoma* antigens conjugated to polyacrylamide beads. Scoring was based on the number of cells binding to polyacrylamide beads, visual evidence of blast transforming cells accompanied by cellular migration, and adherent cell layers surrounding the beads. The maximum reported average scoring always ranged between 3 and 4 (except for one study), even though the index allowed for a score of up to 6 (Table 1). 

More recently, a similar yet more semi-quantitative scoring index was developed, based on quantifying aggregation, defined as a progressive increase in the size (approximate diameter) and specific distribution of cells (further indicated as GI-S, Table 1) [22]. Some of these studies also used beads to induced granuloma formation, leading to an increase in the observed sizes [23]. 

An additional index was introduced by Deknuydt and colleagues, describing the maturation of the formed granuloma (Table 1). The maturation index is similar to granuloma development by size increment—GI-S [24]—but maturation also describes the complexity of the granulomas formed [25]. 

Another index, known as multinucleation index was established with a focus on MGCs. The index is defined as the number of nuclei within MGCs over the total number of nuclei counted across cells per culture [26]. Long-term exposures can further distinguish smaller multinucleated cells (MCs; 2 < number of nuclei < 7) from MGCs (number of nuclei > 15) [27]. Intermediate multinucleated cells (7 < number of nuclei < 15) were, however, not reported. Regardless, an increased multinucleation index quantitatively validates maturation of macrophages and development of granulomas.

Several other studies define granuloma formation through size and number of granulomas and used non-standardized in-house systems to compare across different activators [28] and donors [29]. Accounting for number of granulomas formed provides an additional element towards characterizing granuloma development—an aspect that is currently not addressed with existing granuloma indices. 

### 3.3. Maturation of Granulomas

**Figure 3:**
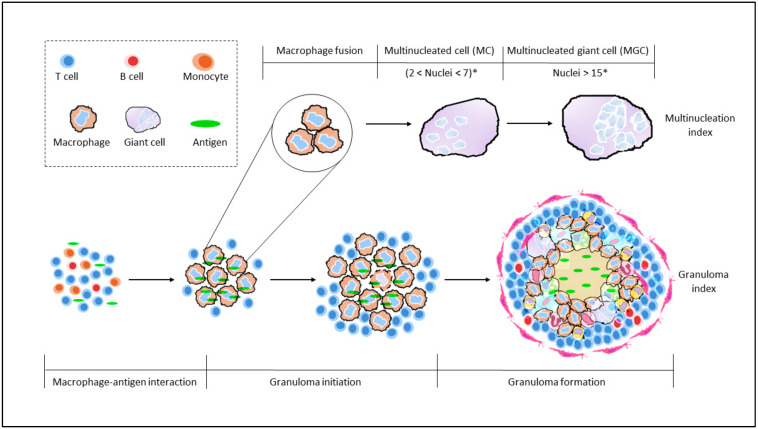
Comparing granuloma indices with maturation of giant cells. Staging of granuloma development is currently still being explored with the already established granuloma indices, GI-B and GI-S. Both GI, however, still lack consideration for monocytes/macrophage maturation. This is countered by the Multinucleation index, wherein an increasing nuclei count is an indication of maturation of macrophages towards the formation of MGC. A combination of these two indices would prove to be more inclusive and well-rounded. * number of nuclei observed would vary, depending on length of exposure.

Though the established scoring systems GI-B and GI-S were successful in describing the development of granulomas, it is however limited in addressing maturation. This is countered by multinucleation index, whereby increased index value is observed typically in the later stages of granuloma development (Figure 3).

**Figure 4:**
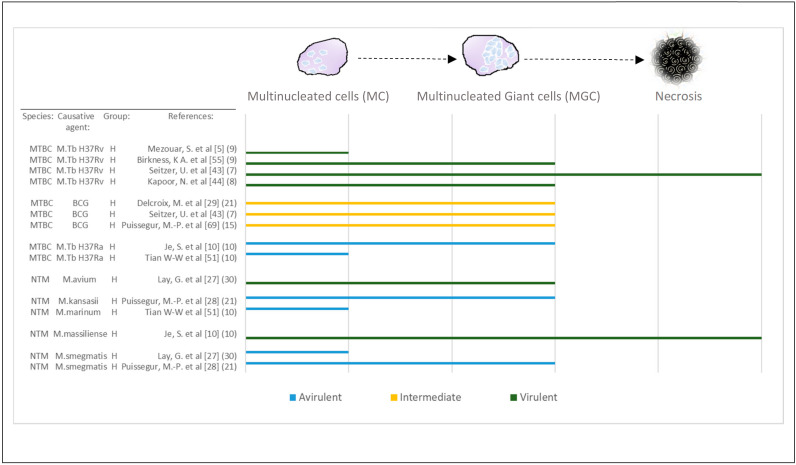
Virulence and late stages of macrophage maturation. Studies that confirmed the presence of MGCs have been selected and recategorized based on the characterization of multinucleated cells (MCs; 2 < number of nuclei < 7) compared with MGCs (number of nuclei > 15). Virulence of mycobacterial species (Table S2) has been evaluated across some of the included studies. In two of these studies, increased virulence was associated with necrosis observed in vitro and MGC formation was observed with avirulent or intermediate species. The remainder of studies did not manage to observe necrosis in culture but confirmed that virulence of species could be differentiated based on either MC or MGC formation. Abbreviations—MTBC: *Mycobacterium tuberculosis* complex; NTM: non-tuberculous mycobacterial species, H: healthy; BCG: Bacillus Calmette–Guérin. [Reference number] per study are included in brackets. Number in brackets refer to length of exposure (days) per study.

Macrophages were also observed to differentiate into MGCs with the exposure to more virulent *M.tb* strains (Table S2), whereas poorly virulent species only induced the formation of MCs [27]. This observation was replicated across several studies (Figure 4). 

#### Lymphocyte Transformation Test (LTT)

On top of morphologically assessing the development of granulomas, 17% of studies also determined the extent of lymphocyte transformation with the incorporation of ^3^H-thymidine [40]. Increased lymphocyte transformation is denoted by the increment of thymidine incorporation induced by respective antigens compared with control conditions [40]. The use of the lymphocyte transformation test (LTT) allows for distinguishing (1) patients who are sensitized from those who are not sensitized, and (2) antigen concentration-dependent degree of lymphocyte transformation. 

In the case of non-infectious antigens, Hanifin and colleagues observed positive transformation in response to beryllium oxide (BeO) only in the Be-sensitized group while transformation was not observed in patients who were unsensitized. When comparing with granulomas in vitro, it was noted that granuloma formation took place in patients who were sensitized as well as non-sensitized; however, a lower concentration of beryllium was sufficient to cause formation of granulomas in patients who are sensitized [40]. 

The studies using *Schistosoma* antigen [33,34,37] confirmed this observation as they reported an increase in GI-B with increased lymphocyte transformation. 

The lack of an increase in response to antigens in LTT was mostly accompanied by a reduced degree of granuloma formation, and likewise, any significantly increased response to antigens in LTT was accompanied by an increased degree of granuloma formation.

The transformation activity of lymphocytes was also assessed with a range of concentrations. With BeO, concentrations from 0.1 to 1 µg/mL [40] were tested, and likewise, Soruri and colleagues tested purified protein derivative (PPD) concentrations ranging from 25 to 400 µg/mL [41]. Both studies [40,41] highlighted that too low concentrations produced low transformation and higher concentrations showed stronger transformation activity; however, in the case of BeO, higher concentrations also induced toxicity, leading to cell destruction.

### 3.4. Cell Surface Markers

**Figure 5:**
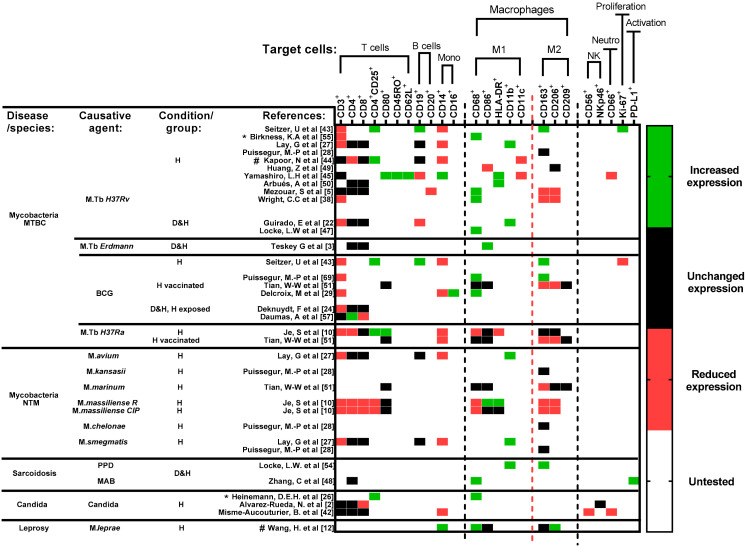
Cell surface markers’ expression profile of included studies. Studies are sorted horizontally according to disease and choice of antigens. Studies focused on mycobacterial diseases are further sorted top-down, based on the virulence of respective strains/species (Table S2). Due to the differences in data presentation and study populations included in each study, any change in expression reflects a significant difference (*p* < 0.05) observed in their respective studies, when compared with either an unexposed condition or directly with a healthy control. Untested (white) refers to markers that were not included per study. Timepoint of comparison across results is always relative to the end of experiment determined in each study. Abbreviations—MTBC: *Mycobacterium tuberculosis* complex; NTM: non-tuberculous mycobacterial species.; BCG: Bacillus Calmette–Guérin; PPD: purified protein derivative; MAB: *Mycobacterium abscessus*; D: diseased; H: healthy; Mono: monocytes; NK: Natural killer cells; Neutro: neutrophils; *: heat-killed strains/species; ^#^: studies that included cell surface markers for microscopic localization and flow cytometric analysis. Reference number per study are included in brackets.

Studies that used cell surface markers either via microscopic localization or flow cytometric analysis through fluorescence-activated cell sorting (FACS) were selected and grouped in a descending order from most used to least used surface marker in accordance with their respective group of target cells (Figure 5). In the case of mycobacterial driven granuloma formation, studies are sorted based on the virulence (see Table S2) of respective strains/species. 

Amongst studies that evaluated granuloma structures via microscopy, a maximum of four surface markers were tested for in 12/14 studies: with a focus on macrophage markers. In studies using flow cytometric analysis, more T, B, monocyte, and macrophage cell markers were used, regardless of choice of agent. In Figure 5, the changes reported are not absolute but relative changes in populations—a change in the proportion/percentage of the marker found when comparing exposed with unexposed condition. 

#### 3.4.1. T Cells

In general, significantly reduced expression of CD3^+^ (total T cells) [10] and a generally unchanged population of CD4^+^ (helper T cells) and CD8^+^ (cytotoxic T cells) cells [24,27,42] were found. 

When focusing on activated or differentiated T cell surface markers however, certain observations could be made:

- Significantly upregulated expression of CD4^+^CD25^+^ (Tregs) [10,26,43,44] and CD80^+^ (co-stimulatory signal for T cell activation) [10,45] were observed.
- Ki-67^+^, a non-specific proliferation marker, was significantly increased [43].
- Increased expression of CD45RO^+^ (memory T) and CD62L^+^ (central memory T) [45] was noted.

#### 3.4.2. B Cells

The expression of CD19^+^ or CD20^+^ B cells is usually reported alongside T cells as part of the rim of cells surrounding the encapsulated core of a granuloma [46]. In most instances, the B cell population was observed to fluctuate in accordance with the total T (CD3^+^) cell population [5,22,44]. 

#### 3.4.3. Monocytes

A significantly reduced expression of the monocyte surface markers CD14^+^ and/or CD16^+^ was observed, indicating monocyte maturation [27,42,43,44]. 

#### 3.4.4. Macrophages

With microscopic localization, specific M1 and M2 macrophage markers such as CD68^+^ and CD163^+^ respectively, were often used as general macrophage markers and not for distinguishing phenotypes [12,26,28,43,44,47,48]. Therefore, these markers confirm the localization of macrophages and are not meant to differentiate the polarized states of macrophages.

The quantification of changes in expression of M1/M2 markers is feasible via flow cytometric analysis since most studies compared the expression levels of both [5,10,12,27,44,45,49,50,51]. 

#### 3.4.5. M1 Macrophages

Significantly increased expression was observed for several markers:

- CD68^+^ expression levels increased, especially when tested with mycobacterial antigens [5,51].
- CD86^+^ and HLA-DR expression levels on macrophages were significantly elevated regardless of the antigen that was used. These markers possibly link the M1 macrophages to antigen presentation and T cell activation [52,53].
- Increased expression of CD11b^+^, a macrophage and MGC marker, was also observed [22,27].

At the same time, a consistent reduction in CD11c^+^ expression was observed across studies [44,45]. 

#### 3.4.6. M2 Macrophages

The following changes in expression were reported for M2 macrophage surface markers in mycobacterial granulomas:

- CD163^+^ [28] and CD206^+^ [12] showed an insignificant and significant decrease in expression, respectively.
- CD209^+^ [51] expression remained unchanged.

These changes were mostly observed with mycobacterial antigens, reaffirming the fact that mycobacterial granuloma formation is mediated by a shift favoring M1 polarization. 

#### 3.4.7. M1/M2 Macrophage Polarization

In some studies, an equilibrium between M1 and M2 could be observed, especially in the case of sarcoidosis and leprosy (Figure 1), wherein at least one of M1 and of M2 macrophage marker expression was elevated [12,54]. In the case of mycobacteria infection, CD86^+^ was significantly expressed at the start of the culture and decreased over time, while CD68^+^, CD163^+^ and CD206^+^ were highly expressed after several days [51]. After 9 days of culture, the coexistence between M1 and M2 macrophages when exposed to *M. leprae* [12] was also confirmed. 

In sarcoidosis, an elevated CD11b^+^ and CD163^+^ expression seems to contribute towards early M2 polarization [54]. 

#### 3.4.8. Remarks Concerning the Use of Cell Surface Markers

A comparison across agents could not be made due to the following:

- Changes in expression of selected markers was assessed via either microscopy or flow cytometric analysis.
- A considerable variability of similar markers was used based on the focus of each study.
- None of the *Schistosoma* antigen-focused studies assessed expression of cell surface markers.

### 3.5. Cytokine Secretion Profile

**Figure 6:**
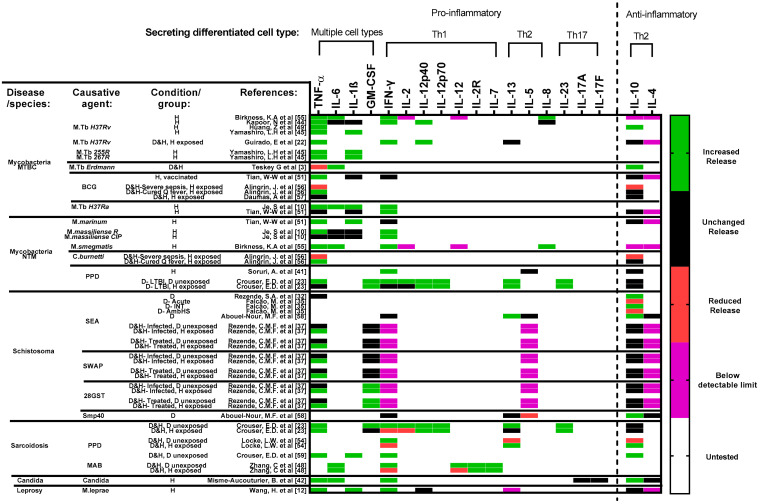
Cytokine secretion profile across included studies. Studies are sorted horizontally according to disease and choice of antigens. Studies focused on mycobacterial diseases are further sorted top-down, based on the virulence of respective strains/species (Table S2). Increased or decreased secretion/release is determined by at most a significant (*p* < 0.05) change observed per cytokine in each study’s results. Untested (white) refers to markers that were not included per study. Several cytokines were tested for but remained below a detectable limit throughout the course of experiments. In this case, since the highlighted cytokines were tested for and not omitted from the respective studies, they have also been included in the figure. Data in such cases are highlighted in purple. Data that focused on the impact of cytokine(s) blockage (Table S3) and/or treatment have been omitted since this figure focuses on antigen-induced granuloma formation only. Timepoint of comparison across results is always relative to the end of experiment determined in each study. Abbreviations—MTBC: *Mycobacterium tuberculosis* complex; NTM: non-tuberculous mycobacterial species.; BCG: Bacillus Calmette–Guérin; PPD: purified protein derivative; SEA: schistosoma egg antigen; SWAP: Adult worm antigen; 28GST: 28-kDa Glutathione S-transferase of *Schistosoma mansoni*; Smp40: S. *mansoni* major egg antigen p40; MAB: *Mycobacterium abscessus*; D: diseased; H: healthy; M: monocytes and/or macrophages; Th: T cells; NK: Natural killer cells; B: B cells; DC: Dendritic cells. Reference number per study are included in brackets. Remark: As cytokines are produced by different cell types, it is to be expected in an in vitro model using PBMCs that the cytokine levels detected are possibly produced by several cells.

In Figure 6, we present a breakdown of the cytokines evaluated via immunoassays, categorized by their respective secreting cells (Th1, Th2 and Th17) as well as by their pro- or anti-inflammatory action. As part of the inclusion criteria, only studies that assessed changes in cytokine secretion profiles are reflected. In mycobacterial granulomas, Th1 pro-inflammatory cytokines were dominant [3,10,22,23,41,44,45,49,51,55,56,57], whereas in *Schistosoma* granulomas, Th2 anti-inflammatory cytokines were most commonly observed [32,35,37,58]. It is however impossible to limit Th1 and Th2 cytokines´ secretion to a single source or cell type; hence, an additional category, multiple cell types, is introduced (Figure 6).

#### 3.5.1. Multiple Cell Types

Different cell types such as lymphocytes, monocytes and NK cells can produce the same cytokines; therefore, the presence of such cytokines cannot be attributed to a specific cell. Most pertinent observations include the following:

- Elevated release of TNF-α was observed in most studies [10,12,22,23,37,42,44,45,49,51,55,56].
- IL-6 changes were inconsistent across studies, with elevation in some [3,10,42,48,55] and no changes [10,44] in others.
- Moderately increased release of IL-1β was observed in most studies [10,12,44,45,51], with differences in the levels observed between mycobacterial species [10,51].
- Changes in GM-CSF levels were observed upon exposure to different antigens: SEA and SWAP against 28GST [37] and between diseased cohorts of sarcoidosis and TB [23].

#### 3.5.2. Th1

An increased release of key Th1 pro-inflammatory cytokine IFN-γ was observed across most studies [10,12,22,23,42,44,48,51,54,55], while IL-2 secretion, although known as a protective cytokine in bacterial control, was reduced in most studies [23]. IL-12 was also barely detectable in the later stages of granuloma formation, resulting in consistently undetectable values across studies [3,55]. IL-12p40 and IL-12p70, subunits of IL-12, were independently assessed, and a significant increase in secretion was noted [22,23,44,51].

#### 3.5.3. Th2 

While IL-5 was not detectable [37] or remained stable [41,58] during the experiments, the chemoattractant IL-8 was elevated from day 1 and a gradual increase in secretion was observed after exposure [55]. IL-13 excretion was very variable between studies; some observed increased release [23,54,58], while others observed no change [22,23,58] or reduced release [54].

#### 3.5.4. Th1/Th2 Balance

In general, there is no clearly defined Th1/Th2 polarization of granulomas in vitro, as the development of granulomatous structure is seemingly regulated by both pro- and anti-inflammatory cytokines [19].

Within most Th1-dominant mycobacterial granuloma models, unchanged secretion levels of IL-10 [22,23,41,51,56,57] were observed while significantly elevated secretion of IL-10 was observed across studies using *Schistosoma* [32,35,58] or *Candida* antigens [42] or in studies focusing on sarcoidosis [54,59]. 

#### 3.5.5. Th17

The modulation of Th17 cytokines were rather inconsistent throughout studies. In most cases, significantly reduced (but unreported) [42] or insignificantly increased expression levels [23] were observed across the Th17 cytokines IL-17, IL-17A and IL-23. 

#### 3.5.6. Changes over Time

Of the studies, 22% selected different time points to evaluate changes in cytokines expression over time. In most studies, however, statistical analysis was not performed across time points. 

**Figure 7:**
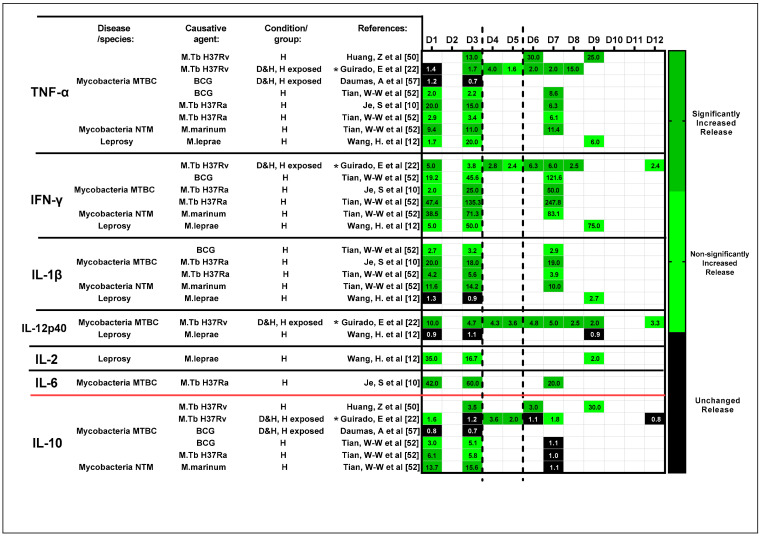
Change in the ratio of the cytokine secretion profile over time. The ratio of cytokine release of the exposed condition (pg/mL)/control condition (pg/mL) was tabulated based on the results presented in the included studies (for completeness, the calculated ratios are included in the colored fields). A three-color grading system was established to track the changes in cytokine release over time (dark green: significantly increased release; light green: non-significantly increased release, but with a ratio larger than 1.5; and black: unchanged release). Studies are organized based on assessed cytokines, which are differentiated as pro-inflammatory—TNF-α, IFN-γ, IL-1β, IL-12p40, IL-2 and IL-6—and anti-inflammatory—IL-10—and further sub-categorized by choice of antigen. Abbreviations—MTBC: *Mycobacterium tuberculosis* complex; NTM: non-tuberculous mycobacterial species; BCG: Bacillus Calmette–Guérin; D: diseased; H: healthy; *: heat-killed strains/species. Reference number per study are included in brackets.

Thus, we tabulated for all relevant studies (6/13) the ratio of the cytokine release of the exposed condition over the control condition per day of evaluation. The ratio tabulated per cytokine in each study was then graded at the different time points. To track the change in cytokine release over time, a color grading system (ranging from green (increased release) over black (unchanged release) to red (reduced release), though a reduced release has not been identified in these studies) was used. A ratio between 0.7 and 1.5 was considered unchanged (black), while dark and light green were used when statistically significant and non-significant differences with ratios larger than 1.5 were reported, respectively. For clarity, the numerical ratios are also included (Figure 7). 

Based on the derived results, a lower ratio of Th1 pro-inflammatory cytokines (TNF-α and IFN-γ) was noted during the early stages of exposure (D1 to D3) while increased ratio of Th2 anti-inflammatory cytokine to IL-10 was observed. This was a common observation across studies regardless of the antigen used. Inconsistent (average difference in ratio across timepoints = 1.47) changes in the secretion of IL-1β and IL-12p40 were also noted across time. In a somewhat later phase, from D6, the increased release of key Th1 cytokines and the reduced release of anti-inflammatory cytokine IL-10 were noted, suggesting a shift from Th2 to Th1 pro-inflammatory responses as granulomas mature in vitro. It is vital to note that limited data for timepoints D4 to D5 impede any conclusion from being drawn. 

## 4. Discussion

In this review, we aimed to identify key parameters that contribute to the genesis and maintenance of human granulomas in vitro. We compared studies focusing on specific granulomatous diseases such as tuberculosis, schistosomiasis and sarcoidosis. We also identified a wide range of experimental conditions and endpoints that were used in these studies. 

The limited availability of models that accurately reflect the progression of granulomatous diseases in humans [23] is an obstacle to better understanding the pathogenesis of granulomatous diseases. Two important elements are noted in this perspective: (i) existing human in vitro models lack the complexity and the chronic host-antigen reactions [60] (Table 2); (ii) maintaining granuloma structures over an extended period of time in vitro, due to the inability to actively recruit cells during development and preservation, is considered a limitation of in vitro models.

However, the PBMC-based in vitro models presented in this review have been able to recapitulate certain key features of human granulomas including—but not limited to—multicellular cytokine profiles, three-dimensional cell structures [23] and confirmation of the identity of cells via cell surface markers (Table 2). Considering this, it should be possible to improve some of the experimental conditions to model in vitro granuloma development. Critical factors not well studied include cell density, accounting for virulence or toxicity of antigens and combining experimental endpoints to assess/score the established granulomas.

Overall, we identified the following main endpoints: (1) granuloma scoring indices, (2) cell surface markers (microscopic evaluation or flow cytometric analysis) and (3) cytokine secretion profiling. Additionally, the common experimental conditions such as choice of antigens and usage of beads are summarized in the infobox. 

### 4.1. Cells/Culture Conditions

A large variation in experimental setups involving different well plates, cell concentration, type of serum and length of exposure has thus far prevented a more in-depth comparative analysis in this review. To accurately evaluate the experimental parameters, the cell concentrations used were recalculated to cell density (cells/cm^2^) and compared against length of exposure (Figure S1A) and day granuloma formation was first observed (Figure S1B). An inverse relationship between a lower cell density range of 3.16 × 10^5^–5.36 × 10^5^ and a longer exposure period of 10–30 days was observed across most studies, irrespective of antigen choice and disease of interest (Table S1).

Regardless, experimental conditions, such as cell density and length of exposure to optimize granuloma formation have not been rationalized in the included studies. For instance, Reyes and colleagues chose to work with a relatively low cell density and observed granuloma formation within a day, although granulomatous structures persisted only for 4 days [68]. Likewise, the benefits of using beads have not been compared or addressed thus far. In the case of tuberculosis, however, mycobacteria-coated beads have been reported to induce well-defined granulomas, just as with direct exposure of live mycobacteria [69]. Consistency in growth rates with or without beads were also observed [27]. 

Serum factors such as cytokines and chemokines might be increased in human serum (HS), allowing for the recognition of unique characteristics that are individualized [48]. However, the comparison of fetal bovine serum (FBS) versus HS has not been assessed thus far.

An overview of the experimental parameters in accordance with each study is also included (Table S1). 

### 4.2. Indexing Granuloma Development

Despite the lack of standardization, the comprehensive outline of the highlighted granuloma indexes is highly reproducible across studies. These indices simplify tracking of granuloma development via their respective staging/scoring systems. 

However, there are still certain weaknesses with the indices:

- Across all indices, a specific threshold is lacking to differentiate clusters of cells from the formation of (well-defined) granulomas. Exposing cells of healthy controls, an index range of 2-2.5 was observed with GI-B [35] and GI-S [22], while a score of 3-4 is commonly observed with GI-B in infected persons.
- Taking into consideration that the maximum score of 5 or 6 with respect to granuloma index GI-B and GI-S were rarely observed, it raises the question of whether increasing the length of experiment after exposure might give rise to a more well-defined granuloma.
- Both indices currently lack definition on the number of granulomas to be expected after exposure.
- A dose-dependent increase in the number of granulomas has thus far only been reported with non-infectious antigens [25], independently from GI.
- GI is only focused on the overall structural evolution of granuloma and does not consider, e.g., the maturation of macrophages to MGC.

### 4.3. Relationship between LTT and Granuloma Index

The pairing of morphological granuloma development with LTT was performed in several studies. Amongst studies that implemented both LTT and GI-B index assessment (7%), a 2–10% increase with thymidine incorporation was met with an increase in score to 2–4 in the GI-B index, more specifically in diseased cohorts [33,37]. The concomitant increase in GI scoring in this case suggests for a T-cell mediated response that assists with granuloma development.

LTT, however, is currently weakly explored in conjunction with human in vitro granuloma modelling, and it could prove to be crucially relevant if a combination of both methods garners further attention.

### 4.4. Cell-Specific Markers

The microscopic localization of select cell surface markers alone has succeeded in confirming the cellular organization of in vitro granulomas as being relevant to human granulomas [27]. At the same time, flow cytometric analysis of granuloma cell populations provided additional insights on which specific cell types contributed a larger role towards granuloma development. For instance, reduced expression of monocytes surface markers is always accompanied by increasing expression of macrophage surface markers, highlighting a shift towards later stages of granuloma development and maturation.

The prevalence of M1/M2 markers is more well addressed when FACS is performed, unlike with microscopic observation, e.g., with the use of either CD68^+^ (M1) and CD163^+^ (M2), structures can be visually confirmed, but the overlapping expression of both markers does not discriminate between M1 and M2.

Although the number of studies that explored the roles of Tregs remains limited [10,43,44], an overall increase in the percentage of CD4^+^ CD25^+^ T cells was observed in granulomatous structures from stimulated cultures when compared with unexposed cultures in healthy controls. It however remains unclear if Tregs in vitro induces a more preventive—by downregulating proliferation of effector T cells—rather than curative effect on inflammatory processes [62].

### 4.5. Cytokine Secretion Profiles

Despite the consensus that TB granulomas are Th1-dominant, several observations show mixed pro-inflammatory expression. Specifically of interest is the insignificant rather than reduced expression observed with Th2 anti-inflammatory cytokine, IL-10. 

Strong IL-10 secretion was observed at early stages of mycobacterial exposure alongside TNF-α, suggesting an acute volatile balance between Th1 pro-inflammatory and Th2 anti-inflammatory responses as well as an IL-10-mediated early protective response against TB [51]. This observation is further validated via tracking of cytokine secretion profiles over time (Figure 7). 

Changes in secretion levels of pro-inflammatory cytokines: IFN-γ and TNF-α can regulate the clinical outcome of the disease, but it is unclear which one is predominant. Some studies indicate that IFN-γ is required for preventing reactivation of LTBI, suggesting an early phase response, while TNF-α seems to contribute towards maintenance of granuloma structures and eventual clearance of antigens, which could be indicative of an early to late phase response [23]. 

In view of cytokine secretion profiles, Th1/Th2/Th17 are to some extent interconvertible [70]. Moreover, defining a cytokine as typical Th1 or Th2 can further limit interpretability. 

Increasing attention should hence be given towards accounting for changes in secretion profiles of sets of cytokines prior to an overall comparison of Th1 with Th2 to account for differences across diseases within the same spectrum. 

### 4.6. Limitations of Our Study

The studies selected for this review focuses on using PBMC-based in vitro models. Hence, the findings in this review might be perceived as limited to specific granulomatous diseases such as tuberculosis. The inclusion of older studies with somewhat different standards in reporting prevents a comparison with other/newer studies. Due to the relative high number of studies focusing on mycobacteria antigens and other infectious antigens, it is still unclear whether the model presented is also transferable for granuloma formation of non-infectious antigens.

### 4.7. Future Directions

Based on the included studies, it is obvious that the literature is mostly focused on infectious agents, such as mycobacteria and *Schistosoma*. It is vital to point out however that a handful of studies focusing on non-infective agents such as beryllium [40] and AlPO_4_ [25] have equally succeeded with granuloma formation in vitro. However, neither of these studies included a focus on cell surface markers and/or cytokine profiling, which limits a more thorough understanding of how the nature of these non-infective antigens can impact granuloma development. This would be essential to improve in vitro research on sarcoidosis, for which the causative antigens are still not identified. Implementing standardization, hence, can improve the applicability of human in vitro granuloma models to encompass more non-infective antigens in the future. 

Alongside standardization of experimental parameters, a refinement of existing granuloma scoring systems should be proposed to include (a) a (mandatory) control cohort as part of the study design; (b) descriptive characterization of cells involved per stage of development (with the use of specific panels of markers in a FACS analysis to pick up more peculiar cell types); (c) size ranges and number of aggregates/granulomas expected per stage of development; (d) staging of macrophage maturation into MGCs to differentiate for MCs (early granuloma) from MGCs (late granuloma); (e) additional stages to account for observed necrosis or dissemination in vitro; (f) an additional stage for disintegration of granuloma over time; and (g) a threshold to distinguish granuloma-like aggregates from a well-defined granuloma.

## 5. Conclusions

The human in vitro granuloma model thus far has been utilized in several ways depending on the area of interest, i.e., testing several antigens, comparing cohorts or as a secondary model to support in vivo data. A more holistic approach to generating granulomas in vitro however still needs to be achieved by first addressing the weaknesses and concerns of the existing model (Table 2). To find a niche in in vitro granuloma modelling, it would be ideal to also focus and report on the daily changes (intracellular and extracellular) rather than at select timepoints—a major limitation in current studies. 

In order to standardize the human in vitro granuloma model and with reference to experimental parameters of included studies, it is suggested to apply a minimum cell density (cells/cm^2^) of >5 × 10^5^ and to incubate cells in the presence of HS.

Depending on the specific aim of the study, the recommended exposure period could range between 5 and 15 days, since 5/8 of schistosomiasis-focused studies chose to end their experiments by day 5, while the 4 studies that focused on BCG alone included a prolonged exposure period between 9 and 21 days.

Additionally, tackling the abovementioned setbacks can then allow this novel and personalized model to be applied across the wider spectrum of granulomatous diseases in the near future.

## 6. Infobox: Important Technical Considerations

### 6.1. Agents

**Figure 8:**
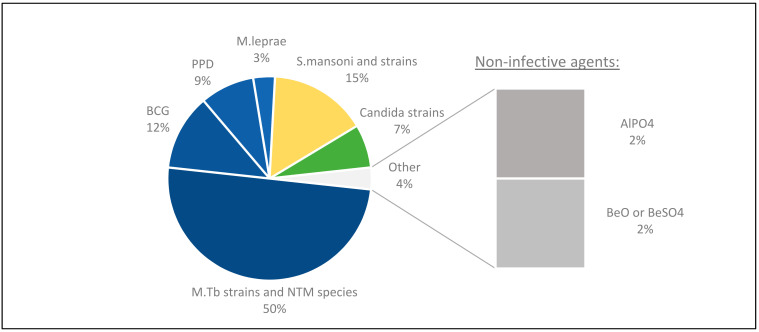
Profiling antigens used in vitro. Most studies focusing on mycobacterial granulomas chose to evaluate different mycobacterial species, and in some studies, BCG and PPD were compared. With other granulomatous diseases, the selection of antigen had specific relevance to either the disease of interest (i.e., *Candida*) or the included patient cohort, especially in the case of non-infective agents.

A range of agents were used to induce granulomas in vitro (Figure 8). 

A majority of the studies were focused on mycobacteria antigens, including several mycobacterial strains such as M. tb H37Rv [5,22,27,28,38,39,43,44,45,47,49,50,55,66,71,72,73,74,75,76], M. tb Erdmann [63,65,77,78], and species such as M. leprae [12,79], M. abscessus [48] as well as BCG [24,29,30,56,57,62,69,80] and PPD [23,54,59,61]. Others used antigens of other infectious species such as Schistosoma [31,32,33,34,35,36,37,58] or Candida [2,26,42,81].

Non-infective agents such as beryllium compounds [40] and aluminium phosphate (AlPO_4_) [25] were also used in individual studies. 

In 12/24 studies, responses between mycobacteria species were evaluated (Table S1), and 6 of these focused on virulence (Table S2). 

### 6.2. Use of Beads

To facilitate granuloma formation, beads were used in PBMC cultures: 25% of studies chose to conjugate antigens to beads prior to PBMC incubation; 8/15 studies chose polyacrylamide beads for the purpose of SEA conjugation; and 5/15 studies used Sepharose beads, with a preference for mycobacterial species and/or BCG. The remaining two studies selected for polystyrene microsphere beads [23,72]. 

Although none of the Schistosomiasis-focused studies prefaced for their preference of conjugating SEA to beads, it was noted that all of these studies implemented the same method, which was first proposed in 1987 [31].

However, no difference in duration to form granulomas was observed with or without covalent conjugation to beads [27]. 

## Figures and Tables

**Table 1:** Characterization of granuloma-like structures with established indices.

Scoring System	Definition	Reference
Granuloma Index GI-B (Granuloma index-beads supported)	Granuloma formation is initiated and scored in accompany of polyacrylamide beads Cellular reactivity was determined by morphological observation based on the following criteria: the number of cells binding to polyacrylamide beads, visual evidence of blast transforming cells accompanied by cellular migration and adherent cell layers surrounding the beads. Three hundred separate determinations of cellular reactivity were made for each experimental group. A numerical score equivalent to the following classification was assigned to each cell-bead reaction observed: No cells binding to the bead <five cells binding to the bead ≥five cells binding to the bead ≥five cells binding to the bead accompanied by a circumoval mononuclear cell migration and blast transformation Adherent cell layer attached to the bead accompanied by circumoval mononuclear cell migration Multiple cell layers surrounding the bead accompanied by mononuclear cell migration The total score was then summed, and the resultant mean expressed as the granuloma index (GI) ± standard deviation (SD). 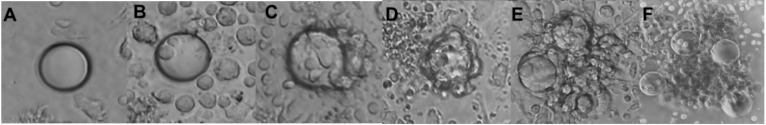 Adapted from Figure 1 of Silva DAAd et al., [30] (A–F refers to score 1 to 6)	[30,31,32,33,34,35,36,37]
Granuloma Index GI-S (Granuloma index-spontaneous)	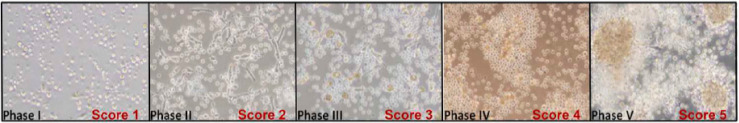 Adapted from Guirado E et al., [23] Granuloma formation in absence of polyacrylamide beads **Phase I** (Score 1): No cellular aggregation. **Phase II** (Score 2): Cellular gathering begins with the recruitment of mostly monocytes and some lymphocytes, approximate diameter: <25/50 μm. **Phase III** (Score 3): Lymphocyte-like cell recruitment increases, approximate diameter: 25/50–100 µm. **Phase IV** (Score 4): The recruited cells form multi-layers, comprising macrophages and lymphocytes, approximate diameter: 100–200/300 μm. **Phase V** (Score 5): The cellular structure shows signs of cellular differentiation (multinucleated giant cells and numerous pseudopodia at the cell surface) mimicking mature in vitro granulomas, with large, differentiated monocyte/macrophage-like cells surrounded by lymphocytes, approximate diameter: >200/500 μm.	[22,23,38]
Index of maturation	Granuloma formation is scored from small to large size Deknuydt F et al. [24] **Index 1:** small and poorly differentiated structures **Index 2:** larger and slightly differentiated structures **Index 3:** Standard granulomas with a good differentiation of cells **Index 4:** Very large and highly differentiated structures Pineton de Chambrun G et al. [25]: results are represented as the total number of granulomas per well and the percentage of small size (index 1) and big size granulomas (indexes 2–4). Mehta M et al., [39]: clusters with granulomas < 100 µm were classified as small and >100 µm were classified as large granulomas.	[25,26,38]
Multinucleation Index	$\mathrm{Multinucleation}\;\mathrm{index}=\frac{\mathrm{number}\;\mathrm{of}\;\mathrm{nuclei}\;\mathrm{within}\;\mathrm{MGC}\;x\;100}{\mathrm{total}\;\mathrm{number}\;\mathrm{of}\;\mathrm{nuclei}\;\mathrm{counted}}$ A total of 300–500 nuclei per culture were counted.	[26]

**Table 2:** Strengths and challenges of existing human in vitro granuloma models.

Strengths	
**Not infection site specific**	Able to generate hepatic Schistosoma granulomas [32,33,37] and pulmonary TB [22,23,38] granulomas in vitro from PBMCs with the same cellular conditions
**Non-invasive and easily manipulatable**	Method of acquiring PBMCs from whole blood is not only non-invasive and readily available but also easily manipulatable. There are no qualms with testing against several antigens [28] simultaneously due to this availability.
**Longitudinal studies**	Allows for the continual assessment of antigen manipulation and tracking of changes in host cells daily [22]
**Host specificity**	Able to distinguish for differences in hosts’ immune responses from unexposed controls [3,22,23,31,35,48,57,61,62,63,64,65,66] as well as predict and mimic granuloma in vivo [25,47]
**Genetic variability**	Molecular patterns are able to distinguish responses to antigen, across cohorts of interest [23]
**Antigen conjugation**	Conjugation of antigens to beads allows for a spectrum of antigens to be tested in vitro [30,37]
**2-/3-dimensional organization**	Able to replicate spatial organization in three dimensions, as observed in human response [54,67]
**Cell surface markers and cytokines expression**	Observations reported reflect published data in literature by recapitulating development in vitro [49]
**Reactivation/Resuscitation**	Resuscitation of dormant M.tb in granulomas under immunosuppressive conditions can be achieved [44]
**Knowledge applicability**	Can be applied to other endpoints such as lymphocyte transformation assay [33,34,37,40,41] and histological analysis [49]
**Weaknesses**	
**Host–antigen interaction**	Complex interactions observed in humans have yet to be achieved in vitro [60]
**Collagen-matrix models**	Extracellular matrix (ECM) may play a role in host–antigen reaction, and collagen matrices have been included in 16% of highlighted studies [3,29,44,45,50,63,64]. Advantages to granuloma maintenance cannot be assessed.
**Continual cell recruitment**	Lack of continual influx of mononuclear phagocytes and lymphocytes to mimic cell recruitment over extended time of exposure [60]
**Vascularization**	Lack of vascularization observed in vitro with PBMCs due to its cellular makeup and hence has not been addressed thus far [50]
**Opportunities**	
**Comorbidities**	Accounting for comorbidities such as diabetes and smoking can facilitate for differences in cohorts [60]
**Microstructure**	Aggregation of granuloma-like structures can be easily distinguished from unexposed controls since it is antigen dependent [44]
**Sensitization in healthy cohort**	Activation or proliferation in an otherwise healthy individual indicates sensitization, which can be determined in vitro with no direct impact on donor
**Concerns/Future challenges**	
**Modulating infection dosage or antigen concentrations**	Impact of high or low dosage/concentration of pathogens/antigens, respectively, has not been assessed but can provide an insight on acute or chronic exposure that is relevant to sarcoidosis.
**Viability**	Cell viability by the end of experiment needs to be accounted for, to determine if cell–cell interactions are antigen dependent or due to cell death.
**Apoptosis/Necrosis**	Assessment of apoptosis and necrosis was only performed in several studies [10,43,44] and requires added focus.

## Data Availability

Not applicable.

## Supplementary Materials

The following supporting information can be downloaded at: https://www.mdpi.com/article/10.3390/cells11050864/s1, Table S1: List of publications; Table S2: Virulence of mycobacterial species/ strains; Table S3: Studies with a focus on evaluating the impact of cytokine(s) blockage; Figure S1: Substantial difference in cell concentrations as well as the well-size were used across studies.
