# Supplementary material for: Assessment of Experimental Techniques That Facilitate Human Granuloma Formation in an In Vitro System: A Systematic Review
Source: Cells. 2022 Mar 2;11(5):864. doi: 10.3390/cells11050864 (PMC8909410; doi:10.3390/cells11050864)
Supplement: Supplementary file 1 [file cells-11-00864-s001.zip › cells-1567336-supplementary.pdf]

Supplementary material/Annex

Supplementary table S1: List of publications

Disease	Antigen	Other species/ antigens	Cohort	Cytokines	Surface markers	Index	Cell density (cells/cm^2)	Type of serum	Length of exposure	Day of granuloma formation	Reference
Be hypersensitivity	BeO or BeSO4	-	Patient & healthy	N	N	-	-	30% HS	5	5	[42]
Brain Injury	*Bacillus Calmette-Guérin* (BCG)	-	Patient & healthy	N	Y	Index of maturation	5.26x10^5	8% HS	12	12	[24]
Leprosy	*M.leprae*	BCG and PPD	Patient	Y	Y	-	6.25x10^5	10% HS	5	5	[76]
Leprosy	*M.leprae*	-	Healthy	Y	Y	-	1.05x10^5	20% FBS	10	9	[12]
Psoriasis vulgaris	Superantigen SPEA	SEB	Patient & healthy	Y	N	-	5.26x10^4	10% FBS	4	4	[80]
Severe sepsis	BCG or *C.burnetti*		Patient & healthy	Y	N	-	7.81x10^5	10% FBS	9	6	[57]
-	AlPO4	-	Healthy	N	N	Index of maturation	5.26x10^5	7.5% HS	7	4	[25]
-	PPD	-	Patient & healthy	N	N	-	-	30% FBS	6	3	[77]
-	*Candida albicans*	-	Healthy	Y	Y	Multinucleation index	3.13x10^5	10% FBS	6	3	[26]
-	2 *Candida* species	-	Healthy	N	Y	-	5.26x10^5	8% HS	6	6	[2]
-	8 *Candida* species	-	Healthy	Y	Y	-	1.32x10^6	8% HS	6	4	[41]
Sarcoidosis	*Candida albicans*	-	Healthy	N	N	-	3.13x10^5	5% HS	6	6	[79]
Sarcoidosis	BCG	-	Patient & healthy	Y	Y	-	1.05x10^6	8% HS	8	3	[45]
Sarcoidosis	PPD or HSA	-	Patient & healthy	Y	N	GI-S	1.14x10^6	10% HS	7	7	[23]*
Sarcoidosis	PPD	-	Patient & healthy	Y	Y	-	1.14x10^6	10% HS	7	7	[56]
Sarcoidosis	PPD	-	Patient & healthy	Y	N	-	-	10% HS	7	7	[78]*
Sarcoidosis	*Mycobacterium abscessus* (MAB)	-	Patient & healthy	Y	Y	-	1.05x10^6	10% HS	7	7	[50]
Schistosomiasis	*S.mansoni*	SWAP	Patient	N	N	GI-B	9.38x10^5	10% HS	5	5	[32]
Schistosomiasis	*S.mansoni*	-	Patient	N	N	GI-B	9.38x10^5	10% HS	5	5	[31]
Schistosomiasis	*S.mansoni*	-	Patient	Y	N	GI-B	9.38x10^5	10% HS	5	5	[30]
Schistosomiasis	*S.mansoni*	Smp40 antigen	Patient	Y	N	-	-	10% HS	3	3	[59]

Schistosomiasis	*S.mansoni*	-	Patient & healthy	N	N	GI-B	5.26x10^{5}	10% HS	15	5	[29]
Schistosomiasis	*S.mansoni*	-	Patient & healthy	Y	N	GI-B	-	10% HS	6	3	[33]
Schistosomiasis	*S.mansoni*	SWAP	Patient & healthy	N	N	GI-B	9.38x10^{5}	10% HS	7	7	[34]
Schistosomiasis	*S.mansoni*	SWAP & 28GST	Patient & healthy	Y	N	GI-B	9.38x10^{5}	10% HS	5	5	[35]
-	*M.Tb H37Rv* or BCG	-	Healthy	N	Y	-	1.88x10^{6}	10% FBS	7	4	[44]
TB	*M.Tb H37Rv* & species	*M.smeg* & *M.avium*	Healthy	Y	Y	-	2.11x10^{5}	10% HS	9	3	[40]
TB	*M.Tb H37Rv* & species	*M.avium, M.smeg, M.phlei, M.kans, M.Tb Erdmann, M.Tb Beijing, M.microti, M.bovis BCG*	Healthy	N	Y	-	5.26x10^{5}	10% HS	30	14	[27]
TB	*M.Tb H37Rv* & species	*M.kansasii, M. chelonae* & *M.smeg*	Healthy	N	Y	-	5.26x10^{5}	10% HS	21	21	[38]
TB	*M.Tb H37Rv* & species	*M.smeg*	Healthy	N	N	-	5.26x10^{5}	7.5% HS	11	3	[65]
TB	*M.Tb H37Ra, H37Rv* & species	*M.smeg* & *M.phlei*	Healthy	N	N	-	5.71x10^{5}	US	8	5	[66]
TB	*M.Tb H37Ra* & species	*M.mass R* & *M.mass CIP*	Healthy	Y	Y	-	5.26x10^{5}	10% HS	10	7	[10]
TB	*M.Tb H37Rv* & species	INH resistant *255R* & *267R*	Healthy	Y	Y	-	1.88x10^{6}	10% FBS	6	3	[46]
TB/T2DM	*M.Tb* strains	*M.Tb Erdmann*	Patient & healthy	Y	Y	-	3.16x10^{5}	5% HS	15	8	[3]
TB/T2DM	*M.Tb* strains	*M.Tb Erdmann*	Patient & healthy	Y	N	-	3.16x10^{5}	5% HS	15	8	[72]
TB/T2DM	*M.Tb* strains	*M.Tb Erdmann*	Healthy	Y	N	-	1.05x10^{5}	5% HS	8	8	[73]
TB	*M.Tb* strains	*M.Tb Erdmann*	Healthy	Y	N	-	3.16x10^{5}	5% HS	8	8	[74]
TB	*M.Tb H37Ra,* BCG & species	*M.marinum*	Healthy; vaccinated	Y	Y	-	5.26x10^{4}	20% FBS	10	7	[53]
TB	*M.Tb H37Ra* or BCG	-	Healthy	N	N	-	5.26x10^{4}	10% FBS	5	1	[62]
TB/T2DM	BCG & *M.Tb* strain	*M.Tb Erdmann*	Patient & healthy	Y	N	-	3.16x10^{5}	5% HS	15	8	[81]

TB/T2DM	BCG & *M.Tb* strain	*M.Tb Erdmann*	Patient & healthy	Y	N	-	3.16x10^5	5% HS	15	8	[75]
TB	*M.Tb H37Rv* or BCG	-	Patient & healthy	Y	N	-	-	20% HS	11	8	[67]
TB	BCG	-	Healthy; vaccinated	N	Y	-	5.26x10^5	10% HS	15	9	[63]
TB	BCG	-	Healthy; 5 vaccinated	N	Y	-	5.36x10^5	10% FBS	21	11	[39]
TB	BCG	-	Patient & healthy	Y	N	GI-B	7.81x10^5	5% FBS	10	5	[28]
TB	BCG	*C.burnetti*	Patient & healthy	Y	Y	-	7.81x10^5	10% FBS	9	9	[58]
TB	*M.Tb H37Rv*	-	Healthy	Y	Y	-	-	20% HS	8	8	[48]
TB	*M.Tb H37Rv*	-	Healthy	Y	Y	-	5.26x10^5	10% HS	9	3	[51]
TB	*M.Tb H37Rv*	-	Healthy	N	N	-	-	10% FBS	3	3	[68]
TB	*M.Tb H37Rv*	-	Healthy	N	N	-	-	10% HS	25	14	[69]
TB	*M.Tb H37Rv*	mmpL11	Healthy	N	Y	GI-S	1.05x10^6	10% HS	12	7	[36]
TB	*M.Tb H37Rv*	-	Healthy	N	N	-	1.05x10^6	10% HS	7	4	[70]
TB	*M.Tb H37Rv*	-	Healthy	Y	Y	-	1.32x10^6	20% HS	8	6	[52]
TB	*M.Tb H37Rv*	ΔwhiB3, whiB3	Healthy	N	N	-	5.26x10^5	US	9	6	[37]
TB	*M.Tb H37Rv*	-	Healthy	Y	Y	-	-	10% FBS	9	3	[5]
TB	*M.Tb H37Rv*	ΔregX3	Healthy	N	N	-	7.89x10^5	US	9	9	[71]
TB	*M.Tb H37Rv*	-	Patient & healthy	Y	Y	GI-S	1.05x10^6	10% HS	12	7	[22]
TB	*M.Tb H37Rv*	-	Patient & healthy	N	Y	-	1.05x10^6	10% HS	7	7	[49]
TB	PPD	-	Healthy	Y	Y	-	-	2.5% HS	6	6	[43]
TB	PPD	-	Patient & healthy	Y	N	-	-	10% HS	7	7	[78]*
TB	PPD or HSA	-	Patient & healthy	Y	N	GI-S	1.14x10^6	10% HS	7	7	[23]*

Legend: Y= Yes, N= No, HS: human serum; FBS: fetal bovine serum; US: unspecified; PPD: purified protein derivative; and * indicates repeating publication that included 2 separate disease cohorts.

Cell density could not be calculated in some cases, as type of well plates used and/or volume were not specified.

Supplementary table S2: Virulence of mycobacterial species/strains

Slowly growing species (> 7 days)			Rapidly growing species (< 7 days)	
**Virulent**	**Intermediate**	**Avirulent**	**Virulent**	**Avirulent**
M.Tb Beijing (MTBC)	*M.bovis BCG* (NTM)	*M.avium* (NTM)	*M.massiliense* (NTM)	*M.abscessus* (NTM)
M.Tb Erdmann (MTBC)	*M.microti OV254* (NTM)	*M.kansasii* (NTM)		*M.chelonae* (NTM)
M.Tb H37Rv (MTBC)		*M.marinum* (NTM)		*M.smegmatis* (NTM)
		M.Tb H37Ra (MTBC)		*M.phlei* (NTM)

Supplementary Table B: Virulence of mycobacterial species/strains as proposed by Lay G et al., 2007 and Je S et al., 2016. MTBC: *Mycobacterial tuberculosis* complex, NTM: non-tuberculous mycobacterial species

Supplementary table S3: Studies with a focus on evaluating the impact of cytokine(s) blockage

Disease / antigen	Reference	Cytokine(s) and/or treatment:
TB-BCG	(58)	Anti-TNF-α
TB-*M.Tb H37Rv*	(37)	Anti-TNF-α
TB-*M.Tb H37Rv*	(43)	Anti-TNF-α
TB-*M.Tb H37Rv*	(5)	Anti-TNF-α and etanercept
Schistosomiasis	(29)	Immune complexes from chronic intestinal schistosomiasis sera
Schistosomiasis	(52)	Anti-IL-4 & Anti-IL-10
Schistosomiasis	(31)	Anti-GM-CSF & Anti-TNF-α

Supplementary figure S1A

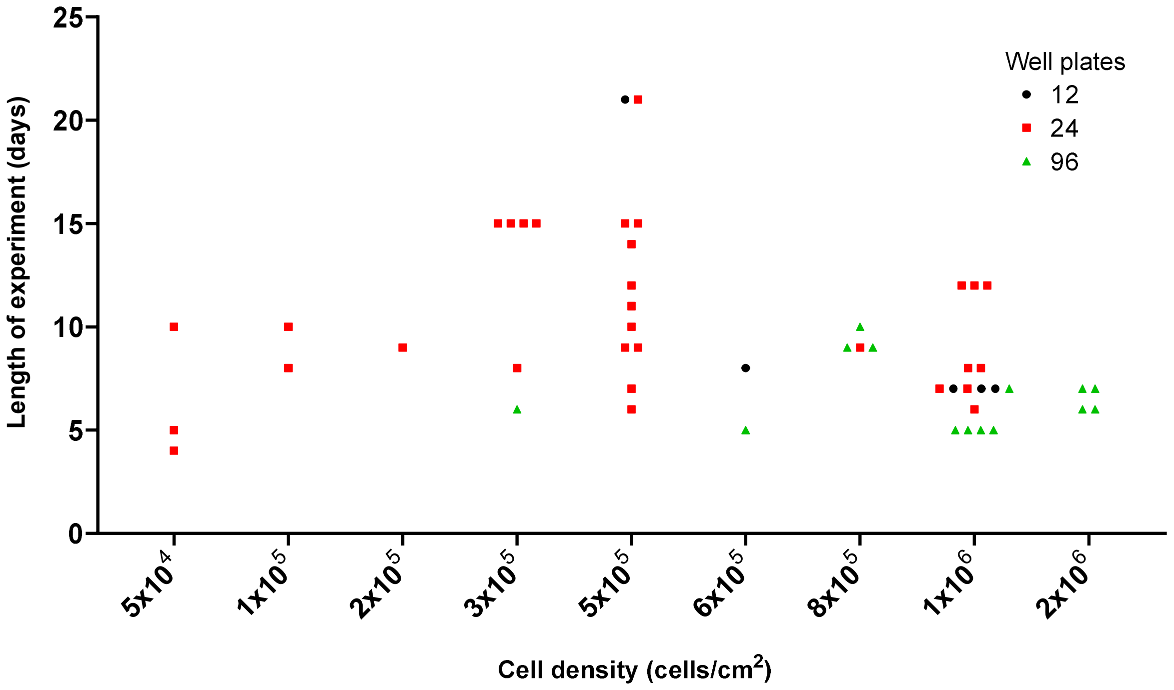

Supplementary figure S1B

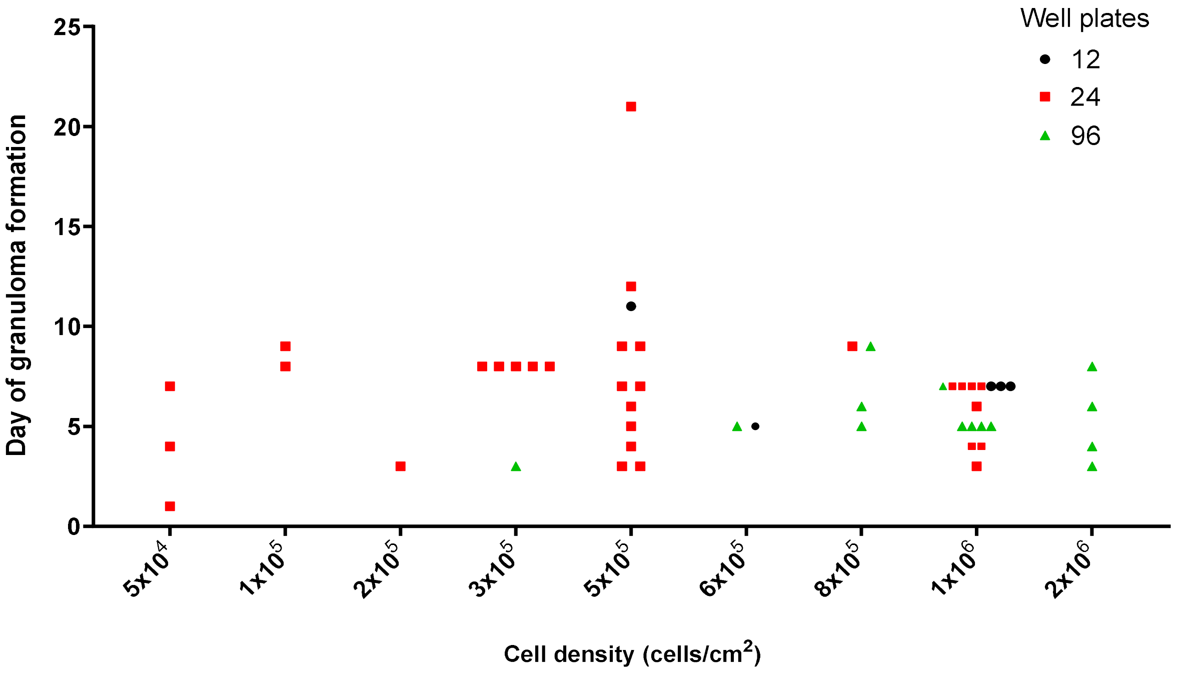

Supplementary Figures S1: Substantial difference in cell concentrations as well as the well-size were used across studies. To compare experimental parameters, cell density per surface area (cells/cm^2) was calculated and compared against length of exposure (**A**) and plotted against day at which granuloma formation was, where applicable, first reported (**B**).
